# Genomic dissection of environmental responsiveness and cross-environment productivity in fenugreek

**DOI:** 10.1371/journal.pone.0361774

**Published:** 2026-10-29

**Authors:** Maha Aljabri, Mustafa M. H. Abd El-Wahab, Amani Mahmoud Dobeie, Mohamed El-Soda

**Affiliations:** 1 Department of Biology, Faculty of Science, Umm Al-Qura University, Makkah, Saudi Arabia; 2 Department of Agronomy, Faculty of Agriculture, Cairo University, Giza, Egypt; 3 Natural Resources Department, Faculty of African Postgraduate Studies, Cairo University, Giza, Egypt; 4 Department of Genetics, Faculty of Agriculture, Cairo University, Giza, Egypt; Arish university, Faculty of agricultural and environmental sciences, EGYPT

## Abstract

Dissecting the genetic basis of genotype × environment interaction (G × E) is important for breeding crops with stable or responsive performance across production environments. Using previously published seed-yield data from 107 Egyptian fenugreek accessions evaluated under two contrasting field environments, we investigated whether yield potential and environmental responsiveness share a common genetic basis. Seed yield per plant was measured in Giza (SYP_G; reference environment) and Wadi El-Natrun (SYP_W; contrasting environment). Nine derived indices were calculated to characterize environmental responsiveness, productivity, and stability: relative performance index (RPI), yield plasticity index (YPI), stress tolerance index (STI), log response ratio (LRR), mean productivity (MP), harmonic mean (HM), tolerance index (TOL), stress susceptibility index (SSI), and AMMI stability value (ASV). The near-zero correlation between SYP_G and SYP_W (r = 0.107) indicated substantial genotype reranking across environments, while several response indices were closely related to SYP_W and therefore predominantly reflected environment-dependent yield expression. Phenotypic analyses also identified contrasting performance strategies, ranging from highly responsive accessions, such as G15 (RPI = 3.629), to highly stable accessions, such as G41 (YPI = 0.001). These phenotypes were integrated with 38,142 SNPs using univariate association-mapping approaches. In addition, SYP_G and SYP_W were subjected to multivariate association-mapping. Association mapping identified one genome-wide-significant SNP for STI and several suggestive loci showed concordant associations across mapping methods. Within the current panel, marker coverage, and significance thresholds, most adaptation-index associations did not overlap previously reported yield loci; however, dDocent_Contig_39939_66, previously associated with seed yield in this panel, also showed cross-method suggestive associations with RPI and TOL. Comparative genomic analysis of three legume reference genomes supported two gene-linked candidate loci associated with STI: a cation/H⁺ antiporter and an importin subunit beta-1. The same analysis classified two of the strongest statistical signals, including the sole genome-wide-significant locus, as repetitive or transposable-element sequences rather than single-copy genes. These findings demonstrate that statistical association strength does not necessarily establish a coding-gene basis for loci identified using reference-free ddRAD markers. Within the current population size and marker coverage, environmental responsiveness and productivity appeared partially distinct but biologically connected components of environment-dependent yield performance. The results provide a genomic and phenotypic framework for prioritizing fenugreek germplasm for further evaluation while emphasizing the need for independent-population, multi-season, and functional validation of the identified candidate loci.

## Introduction

Fenugreek (Trigonella foenum-graecum L.) is an annual self-pollinated legume cultivated throughout the Mediterranean region, the Middle East, and South Asia for its nutritional, medicinal, and agronomic value. Fenugreek is widely used as a source of edible seeds, bioactive compounds, forage biomass, and biological nitrogen fixation, making it an important component of sustainable agricultural systems [1]. In Egypt, fenugreek is traditionally cultivated across diverse agroecological regions, contributing to crop diversification and smallholder livelihoods. Despite increasing interest in improving fenugreek, genomic and omics-based investigations remain limited compared with those in major legume crops, and the molecular basis of environmental adaptation remains poorly understood [1,2]. Therefore, identifying germplasm and genetic loci associated with productivity across environments is a central objective for breeding programmes operating under variable field conditions.

Environmental adaptation—the capacity of a genotype to maintain or improve performance across contrasting environmental conditions—is a central objective of crop improvement programmes. As climate variability increases and cultivation expands into marginal production areas, breeders must identify germplasm capable of maintaining productivity across heterogeneous environments. However, genotype × environment interaction (G × E) often complicates selection because superior performance in one environment may not translate into superior performance elsewhere [3]. Understanding the phenotypic and genomic determinants of G × E is therefore essential for developing climate-resilient cultivars that maintain productivity under variable environmental conditions.

Recent studies in Mediterranean legume systems have emphasized that adaptation results from complex interactions among morphological, physiological, and genetic traits, highlighting the need for integrative genomic approaches to identify adapted germplasm. A fundamental question in adaptation genetics is whether the same genomic mechanisms control environmental responsiveness and productivity, or whether they represent partially independent biological processes [2]. Yield performance and adaptive capacity are frequently treated as synonymous breeding objectives, yet adaptation may involve physiological and developmental responses that are distinct from those directly controlling productivity. Disentangling these components is therefore important for understanding crop resilience and for designing breeding strategies that simultaneously improve yield and environmental responsiveness.

Association mapping has become a powerful approach for dissecting the genetic architecture of complex traits in diverse germplasm collections [4,5]. By integrating high-density molecular markers with phenotypic variation, association mapping enables the identification of loci associated with agronomic performance, stress tolerance, developmental traits, and adaptation-related responses. In legumes, association mapping has been widely applied to investigate drought tolerance, salinity adaptation, yield stability, flowering time, and other traits contributing to environmental adaptation [6–9]. Genomic approaches are increasingly central to the discovery of adaptive alleles and the deployment of climate-resilient germplasm in legume crops [2]. However, comparable studies remain limited in fenugreek.

Recent genomic investigations have established a valuable foundation for fenugreek genetics [10], in which ddRAD sequencing was used to characterise population structure and identify SNPs associated with seed morphometric traits in a diverse panel of Egyptian accessions. Subsequently, the same germplasm panel was employed to identify SNP markers associated with distinctness, uniformity, and stability (DUS) descriptors [8]. More recently, evaluation of this collection under controlled salinity treatments revealed substantial genetic variation in stress-responsive morphological traits and identified SNPs associated with salinity responses, demonstrating that the panel harbours exploitable allelic diversity for environmental responsiveness [9]. Together, these studies indicate that this germplasm constitutes a valuable genomic resource for dissecting complex adaptive traits.

Recent studies have successfully employed stress-tolerance indices as GWAS phenotypes to dissect adaptation under contrasting environments. For example, genomic loci associated with drought tolerance indices were mapped in wheat [11,12]. However, comparable adaptation-focused genomic analyses remain unavailable in fenugreek, particularly under contrasting field environments.

Given the high cost and logistical complexity of conducting multi-environment field trials, particularly in under-researched crops such as fenugreek, maximizing biological inference from existing phenotypic datasets through advanced statistical and genomic frameworks represents a critical strategy for accelerating genetic improvement. Fenugreek exhibits considerable adaptation to drought- and salinity-prone environments, yet the genomic mechanisms underlying environmental responsiveness remain largely unresolved [1].

While seed-yield performance of this panel under the two environments has been reported previously [8], the present study reanalyses these data using adaptation-focused indices and association mapping frameworks to investigate the genetic basis of environmental responsiveness. We investigate environmental adaptation in a panel of 107 Egyptian fenugreek accessions evaluated under two contrasting field environments: Giza, representing a conventional agricultural production environment, and Wadi El-Natrun, representing a contrasting inland environment characterised by distinct edaphic and climatic conditions. To characterize environmental responsiveness, joint productivity, and stability, we derived nine complementary indices: RPI, YPI, STI, LRR, MP, HM, TOL, SSI, and ASV. These indices were integrated with genome-wide SNP data using univariate association mapping, while SYP_G and SYP_W were also analyzed jointly using a multivariate linear mixed model.

Specifically, we address three questions: (i) Does Wadi El-Natrun function primarily as a stress-imposing environment or as an environment that reveals adaptive genetic variation within Egyptian fenugreek germplasm? (ii) Do productivity and adaptation share common genomic determinants or represent distinct genetic architectures? (iii) Which candidate genes may contribute to environmental responsiveness in fenugreek? These findings have direct implications for breeding strategies to improve both productivity and environmental resilience in fenugreek. By combining adaptation-related phenotypes, multi-trait selection indices, association mapping, and comparative genomic validation, this study provides a genomic framework for understanding environmental adaptation in fenugreek. It identifies germplasm resources for future breeding programmes.

## Materials and methods

### Plant material

A panel of 107 Egyptian fenugreek (*Trigonella foenum-graecum* L.) accessions assembled from 15 governorates spanning the major fenugreek-growing regions of Egypt [10] was used in this study. The present study extends that genomic resource to cover environmental adaptation-related indices.

### Field evaluation and phenotypic measurement

Accessions were evaluated in a randomised complete block design (RCBD) with three replicates per environment at two sites: Environment 1 – Giza (30.01 °N, 31.21 °E), representing a reference Nile Delta–adjacent site with fertile alluvial clay loam soils (39% clay, 2.43% organic matter) and relatively moderate climatic conditions, and Environment 2 – Wadi El-Natrun (30.37 °N, 30.33 °E), representing a contrasting inland production environment characterised by sandy calcareous soils (93% sand, 1.67% organic matter), elevated soil salinity (EC 5.37 dS m^−1^), higher evaporative demand, and saline irrigation water (Na ⁺ 10.4 meq L^−1^). These differences create a strong abiotic-stress gradient across environments (S1 Table). The study was designed to evaluate environmental adaptation-related indices across naturally contrasting field environments rather than responses to a defined abiotic stress treatment. The primary phenotype was Seed Yield per Plant (SYP, g), recorded at physiological maturity as the mean total seed mass per individual plant across replicates. Environment-specific values are designated SYP_G (Giza) and SYP_W (Wadi El-Natrun).

The SYP_G and SYP_W data were originally reported by Abd El-Wahab et al. (2023) [8]. In the present study, the existing dataset was reanalysed to derive nine environmental-response, productivity, and stability indices and to investigate their genomic architecture through association mapping.

### SNP genotyping and quality control

After applying a minor allele frequency (MAF) threshold of ≥ 5%, 38,142 markers remained across 107 accessions distributed over 8,814 sequence contigs assembled using the dDocent pipeline [10]. SNP dosage values (0, 1, or 2 copies of the alternative allele) were encoded numerically.

### Derivation of environmental-adaptation related phenotypes

To quantify genotype performance across environments, nine derived indices were calculated from SYP_G and SYP_W.

### Relative performance index (RPI)


$$\begin{array}{c} RPI=SYP\_W/SYP\_G \end{array}$$

RPI > 1 indicates superior Wadi performance; RPI ≈ 1 indicates environmental homeostasis; RPI < 1 indicates Giza specialisation. RPI served as the primary adaptation phenotype.

### Yield plasticity index (YPI)


$$\begin{array}{c} YPI=|SYP\_W-SYP\_G|\text{\textasciitilde{}}/\text{\textasciitilde{}}max(SYP\_W,\text{\textasciitilde{}}SYP\_G) \end{array}$$

YPI is bounded in [0, 1] and quantifies the magnitude of yield change between environments, irrespective of direction, with 0 denoting identical performance and values approaching 1 denoting maximal plasticity.

### Stress tolerance index (STI)


$$\begin{array}{c} STI=(SYP\_G\times SYP\_W)\text{\textasciitilde{}}/\text{\textasciitilde{}}{mean(SYP\_G)}^{2} \end{array}$$

STI [13] rewards genotypes with high, jointly productive performance across both environments; mean(SYP_G) was used as the reference denominator (The Giza mean was retained as the reference denominator. Because Wadi El-Natrun had the higher panel mean yield, STI is interpreted here as an index of joint cross-environment productivity rather than conventional stress tolerance).

### Log response ratio (LRR)


$$\begin{array}{c} LRR=ln(SYP\_W/SYP\_G) \end{array}$$

LRR is the natural-log-transformed, symmetric analogue of RPI, giving a directional, approximately normally distributed measure of proportional site response.

### Mean productivity (MP)


$$\begin{array}{c} MP=(SYP\_G+SYP\_W)/2 \end{array}$$

MP is the simple arithmetic mean productivity across sites [14].

### Harmonic mean (HM)


$$\begin{array}{c} HM=2\times SYP\_G\times SYP\_W/(SYP\_G+SYP\_W) \end{array}$$

HM [13] is an alternative productivity index that, relative to MP, down-weights genotypes with a large imbalance between the two site yields.

### Tolerance (TOL)


$$\begin{array}{c} TOL=SYP\_W-SYP\_G \end{array}$$

TOL [14] is the signed absolute yield difference between environments.

### Stress susceptibility index (SSI)


$$\begin{array}{c} SSI=(1-SYP\_G/SYP\_W)/(1-mean(SYP\_G)/mean(SYP\_W)) \end{array}$$

SSI [15] expresses each genotype’s proportional yield reduction in Giza relative to Wadi, normalized by the panel-average proportional reduction; SSI ≈ 1 indicates average susceptibility, SSI < 1 indicates below-average susceptibility (relative tolerance), and SSI > 1 indicates above-average susceptibility.

### AMMI stability value (ASV)


$$\begin{array}{c} ASV=(1/\surd 2times|(SYP\_G-mean(SYP\_G))-(SYP\_W-mean(SYP\_W))| \end{array}$$

ASV was computed following the additive main effects and multiplicative interaction (AMMI) approach [16], applied here to the reduced two-environment genotype × environment matrix, for which the interaction term has a single (IPCA1) axis; ASV summarises the magnitude of each genotype’s environment-specific deviation from its main effect, with higher values indicating greater G x E (lower stability) across the two sites.

### Phenotypic quality control and distributional analysis

Normality was assessed using the Shapiro–Wilk test. Phenotypic distributions were characterised by mean, median, standard deviation (SD), coefficient of variation (CV), minimum, maximum, skewness, and excess kurtosis. Pairwise Pearson and Spearman correlations were computed among the two environment-specific yield traits and the nine derived indices.

### Environmental comparison

The mean yield difference between environments was assessed using a paired Student’s *t*-test and the Wilcoxon signed-rank test. Pearson (r) and Spearman (ρ) correlations between SYP_G and SYP_W were computed to quantify G × E interaction.

### Elite germplasm classification

#### Multi-trait genotype-ideotype distance index (MGIDI).

The MGIDI [17] was computed with the mgidi() function of the R package metan v1.18.0 [18]; R Core Team, 2024, R v4.3.3). Each index was rescaled to a 0–100 scale so that a value of 100 represented the desired selection direction. A factor analysis was performed on the rescaled data using principal component extraction, retaining factors with eigenvalues ≥ 1 (Kaiser’s criterion), followed by varimax rotation. Factor scores were estimated for each genotype and for a theoretical ideotype possessing the ideal value (100) for every index. The MGIDI was calculated as the Euclidean distance, in the retained factor space, between each genotype and the ideotype; smaller MGIDI values indicate genotypes closer to the ideotype. A selection intensity of 15% (16 of 107 genotypes) was used, and the selection differential (SD) and percent SD were computed for each index by comparing the mean of the selected genotypes with the overall mean.

### Factor analysis and ideotype-design index (FAI-BLUP)

The FAI-BLUP index [19] was computed with the fai_blup() function of metan, using the same 15% selection intensity and directional assumptions as above (via the DI/UI arguments). Indices were standardized by their standard deviation, and the same factor-extraction and varimax-rotation procedure described above was applied. For each retained factor, a desirable (D) and undesirable (U) reference value was defined from the extreme (maximum or minimum, depending on the selection direction) standardized value observed among genotypes for the indices loading on that factor. All 2^k combinations of D/U across the k retained factors were used to build k candidate ideotypes; the fully desirable ideotype (ID1, all factors at their D value) was taken as the reference. The spatial probability of each genotype resembling ID1 was calculated from the standardized Euclidean distance between the genotype and each candidate ideotype (FAI-BLUP index); higher values indicate closer resemblance to the ideal genotype.

### Association mapping

#### Univariate association mapping.

Association mapping was performed independently for each of the 11 traits using three complementary models, chosen to capture single-locus, multi-locus, and iterative fixed-effect approaches to association testing:

**Genome-wide Efficient Mixed Model Association (GEMMA):** The linear mixed model (LMM) implemented in GEMMA v0.98.5 [20] was used as the single-locus baseline. A centred genomic relatedness (kinship) matrix was first estimated from the full set of genotyped SNPs (GEMMA -gk 1). For each trait, the model y = Wα + xβ + u + ε, u ~ N(0, σ²ₐK), ε ~ N(0, σ²_e_I) was fitted at each SNPe W war, whes an intercept-only covariate matrix, x the genotype dosage at the candidate SNP, and u a random polygenic effect with covariance proportional to K. Association was assessed using GEMMA’s likelihood-ratio-based framework (-lmm 4); the Wald test p-value (p_wald) was used as the primary statistic.

**Multi-Locus Mixed Model (MLMM).** MLMM [21], as implemented in the R package GAPIT v4.1.0 [22], was used to account for multiple independent loci simultaneously. MLMM begins from the same kinship-corrected mixed-model framework as GEMMA but performs a forward-backwards stepwise search, iteratively introducing the most significant SNP as a fixed-effect cofactor into the model and re-estimating variance components at each step (forward phase), then removing cofactors that no longer contribute significantly (backward phase). Population structure was controlled using GAPIT’s internally computed Zhang kinship matrix together with the first three principal components of the marker data as fixed-effect covariates.

**Bayesian-information and Linkage-disequilibrium Iteratively Nested Keyway (BLINK).** BLINK [23], also implemented in GAPIT v4.1.0, was used as a third, methodologically distinct approach. Rather than correcting for relatedness via a kinship matrix, BLINK iteratively selects associated markers and re-tests the genome using those markers as fixed-effect covariates, removing markers in linkage disequilibrium with already-selected markers between iterations. The first three principal components were again supplied as fixed-effect covariates.

### Multivariate association analysis

SYP_G and SYP_W were modelled jointly using GEMMA’s multivariate LMM (-lmm 1), which extends the univariate model described above to a single random-effects covariance structure shared between the two phenotypes (genetic and residual variance-covariance matrices, estimated once by REML under the null) and reports a two-degree-of-freedom Wald test per SNP for the joint hypothesis that both phenotype effects are zero. The kinship matrix and PC1–3 covariates were held fixed across all markers tested.

Bonferroni thresholds were calculated separately for each model as 0.05 divided by the number of SNPs tested. FDR significance was assessed independently within each trait–model scan using the Benjamini–Hochberg procedure at q ≤ 0.05. Associations with nominal P < 1 × 10^−4^ were classified as suggestive candidate associations. Genomic inflation was assessed by λ_GC_ [24]. λGC was used solely as a diagnostic metric and not for p-value adjustment.

### Homology-based candidate gene annotation

This analysis proceeded in three stages. First, the 101 bp flanking sequence of the single strongest signal in the dataset was searched against the NCBI core_nt and nr databases using web-based BLASTn, and predicted mRNA hits with an intact open reading frame were retrieved as full GenBank records. Second, translated protein sequences from these hits were submitted to SMART for conserved-domain identification, and a pairwise local alignment (Biopython Bio. Align, BLOSUM62) was performed between independently predicted, taxonomically distant homologs. Third, NCBI BLAST+ v2.12.0 was used to search all 32 candidate-SNP flanking sequences locally against four independently downloaded reference datasets: *Medicago truncatula* (GCF_003473485.1, diploid, genome + annotated transcripts), *Trifolium repens* (GCA_030408175.1, allotetraploid, genome only), *Trifolium pratense* (GCF_020283565.1, diploid, genome only), and the fenugreek plastid and mitochondrial genomes (NC_042857.1, NC_072135.1; no nuclear reference genome currently exists for fenugreek). blastn was run with -task blastn -word_size 11 -evalue 10 (word size 7 for the small organelle database).

A hit was classified as strong/reliable only if it met all three pre-declared criteria, fixed before any results were examined: alignment length ≥ 30 bp, per cent identity ≥ 85%, and E-value ≤ 1 × 10^−5^. For each SNP, strong hits within 500 bp of one another on the same chromosome/scaffold were merged into a single locus, and the number of distinct genomic loci was used as a direct, assembly-based measure of copy number: single-copy or small-tandem-cluster loci were interpreted as candidate genes, whereas sequences recovered at many dispersed genomic positions were interpreted as repetitive elements. Because *T. repens* is a natural allotetraploid, a genuine single-copy nuclear gene was expected to appear as two loci (one per subgenome) rather than one.

## Results

### Phenotypic variation in seed yield

All 107 accessions had complete phenotypic records. SYP_G ranged from 34.0 g (G122) to 213.9 g (G39), with a panel mean of 102.22 ± 32.44 g (CV = 31.7%) and an approximately normal distribution (Shapiro–Wilk W = 0.982, p = 0.155; skewness = 0.506; Table 1). In contrast, SYP_W showed substantially greater variation, ranging from 29.1 g (G10) to 409.0 g (G15) (mean = 148.91 ± 74.83 g; CV = 50.3%), with significant right skew (W = 0.905, p < 0.001; skewness = 1.259).

**Table 1:** Descriptive statistics of seed yield and adaptation indices across environments. Summary statistics (mean, median, standard deviation [SD], coefficient of variation [CV], minimum [Min], maximum [Max], SD: standard deviation; CV: coefficient of variation, and Shapiro–Wilk test p-values [SW p]) for seed yield and derived adaptation indices. SYP_G: seed yield per plant in Giza; SYP_W SYP in Wadi El-Natrun (SYP_W); RPI: relative performance index; YPI: yield plasticity index; STI: stress tolerance index; LRR: log response ratio; MP: mean productivity; HM: harmonic mean; TOL: tolerance index; ASV: AMMI stability value; SSI: stress susceptibility index.

Trait	Mean	Median	SD	CV (%)	Min	Max	Skewness	SW p
SYP_G (g)	102.22	101.2	32.44	31.7	34	213.9	0.506	0.155
SYP_W (g)	148.91	128.9	74.83	50.3	29.1	409	1.259	<0.001
RPI	1.576	1.334	0.868	55.1	0.323	4.56	1.283	<0.001
YPI	0.359	0.361	0.209	58.2	0.001	0.781	0.152	0.011
STI	1.481	1.288	0.915	61.8	0.12	4.74	1.381	<0.001
LRR	0.313	0.288	0.545	174.3	−1.131	1.517	−0.174	0.865
MP (g)	125.57	117.9	42.34	33.7	35.45	260.85	0.863	0.001
HM (g)	113.17	109.49	33.06	29.2	35.39	206.23	0.302	0.586
TOL (g)	46.68	31.2	78.32	167.8	−142.5	296.3	0.861	<0.001
ASV	42.12	33.18	35.73	84.8	0.766	176.51	1.577	<0.001
SSI	0.475	0.799	1.658	349.2	−6.697	2.49	−1.96	<0.001

Among the adaptation and productivity indices, RPI ranged from 0.323 to 4.560 (mean ± SD = 1.576 ± 0.868) Whereas YPI ranged from 0.001 to 0.781 (mean = 0.359 ± 0.209. STI showed substantial variation across the panel (0.120–4.740; mean = 1.481 ± 0.915), MP ranged from 35.45 to 260.85 g, and HM from 35.39 to 206.23 g. TOL exhibited a wide distribution (−142.5 to 296.3 g), LRR displayed an approximately symmetric distribution (mean = 0.313; skewness = −0.174; Shapiro-Wilk p = 0.865), whereas ASV and SSI showed substantial non-normality and broad phenotypic ranges. LRR and HM were the only derived indices that were consistent with normality (Shapiro-Wilk p > 0.05), while most adaptation indices showed significant departures from normality and positive skewness

### Pairwise trait correlations

Pearson correlations among yield traits (SYP_G and SYP_W) and adaptation-related indices (Fig 1) revealed two major patterns. Yield under Wadi El-Natrun (SYP_W) was strongly associated with adaptation metrics such as RPI, LRR, TOL, STI, and MP, whereas yield under Giza (SYP_G) showed weak or negative correlations with several adaptation indices. Strong associations were observed among RPI, LRR, and TOL, while ASV exhibited relatively weak correlations with most traits.

**Fig 1:**
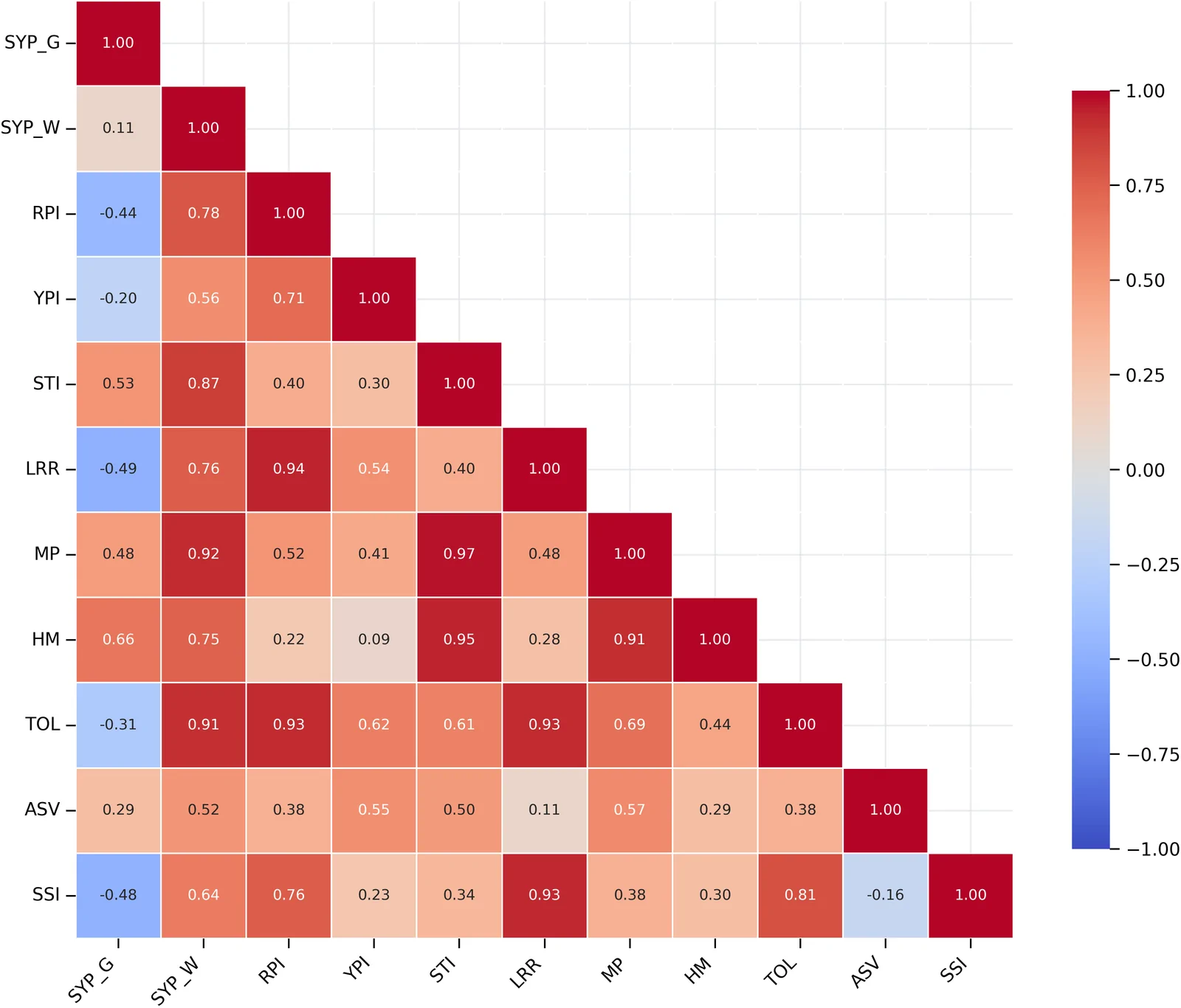
Pearson correlation matrix among yield traits and adaptation-related indices in the Egyptian fenugreek diversity panel. Correlation coefficients are shown for seed yield per plant in Giza (SYP_G) and Wadi El-Natrun (SYP_W), relative performance index (RPI), yield plasticity index (YPI), stress tolerance index (STI), log response ratio (LRR), mean productivity (MP), harmonic mean (HM), tolerance index (TOL), AMMI stability value (ASV), and stress susceptibility index (SSI). Cell colours represent the magnitude and direction of correlations, ranging from strong negative (blue) to strong positive (red) associations. Strong positive relationships were observed among several adaptation and stress-response indices, particularly between RPI and LRR (r = 0.94), RPI and TOL (r = 0.93), and STI and MP (r = 0.97). In contrast, seed yield under Giza conditions (SYP_G) showed negative correlations with adaptation-related indices such as RPI, LRR, and SSI, highlighting contrasting genotype performance across environments.

### Environmental comparison

Mean SYP_W (148.91 g) exceeded mean SYP_G (102.22 g) by 46.69 g per plant (+45.7%). Both the paired *t*-test (t _(106) = −6.166, p < 0.001) and the Wilcoxon signed-rank test (W = 1,128, p < 0.001) confirmed that_ the difference was highly significant. Seventy-nine accessions (73.8%) had RPI > 1; 28 (26.2%) had RPI < 1.

### Elite germplasm identification

Factor analysis of the nine stress-tolerance indices retained three factors with eigenvalues ≥ 1, jointly explaining 95.8% of the total variance (FA1 = 59.1%, FA2 = 22.1%, FA3 = 14.7%; mean communality = 0.96). Trait grouping after varimax rotation was biologically coherent: FA1 was defined by the susceptibility/gap-type indices RPI, LRR, TOL and SSI (loadings from −0.85 to −0.98); FA2 by the productivity-type indices STI, MP and HM (loadings from 0.89 to 0.98); and FA3 primarily by YPI and ASV (loadings of 0.81 and 0.87, respectively).

MGIDI. At 15% selection intensity, 16 genotypes were selected as superior multi-trait ideotypes (lowest MGIDI, range 3.19–4.51): G5, G37, G41, G25, G45, G3, G59, G17, G109, G26, G22, G51, G43, G105, G6 and G34. The selection differentials indicated coordinated improvement across all three factors: FA1-related indices decreased substantially in the selected group (RPI: −30.0%, LRR: −88.6%, TOL: −70.2%, SSI: −112.4%), while FA2-related indices increased (STI: + 55.6%, MP: + 23.4%, HM: + 32.3%). FA3-related indices showed a smaller, mixed response (YPI: −39.8%; ASV: + 6.8%, in the undesired direction), identifying FA3 as the weakest-gain factor under MGIDI-based selection.

FAI-BLUP. The same selection intensity identified 16 genotypes with the highest probability of resembling the fully desirable ideotype (FAI-BLUP range 0.184–0.121): G5, G41, G37, G45, G25, G3, G59, G17, G22, G51, G105, G109, G26, G34, G101 and G123. Selection differentials followed the same pattern observed for MGIDI (FA1 indices reduced by 25.7–64.2%, TOL and SSI by 49.6% and 53.3%; FA2 indices increased by 24.6–58.4%), and in this case ASV also showed a favorable decrease (−9.3%) in the selected group.

Agreement between indices. Fourteen of the sixteen genotypes selected by MGIDI were also selected by FAI-BLUP, corresponding to a coincidence index of 87.5% (G5, G37, G41, G45, G25, G3, G59, G17, G109, G26, G22, G51, G105 and G34 selected by both methods). This high level of agreement supports the robustness of the multi-trait ranking and indicates that either index can be used with confidence to identify cross-environment productive genotypes combining favorable yield potential (FA2) with a reduced yield gap between the two contrasting production environments (FA1).

### Association mapping analysis

#### Univariate association analysis.

Association analysis was performed for all 11 phenotypes using three independent methods (GEMMA univariate LMM, MLMM, and BLINK). At the strict, genome-wide-corrected significance thresholds (Bonferroni and FDR ≤ 5%), only one trait produced any significant association: stress tolerance index (STI), for which a single SNP, dDocent_Contig_31206_12 (contig 31206; marker-order position 5995 in the concatenated marker display), was significant in all three methods (GEMMA p = 5.8 × 10^−7^; MLMM p = 4.6 × 10^−7^; BLINK p = 3.0 × 10^−9^). No other trait produced a Bonferroni- or FDR-significant association in any of the three methods (Table 2).

**Table 2:** Cross-method-concordant candidate SNPs identified by all three association mapping approaches, namely GEMMA, MLMM, and BLINK, for environmental-response, productivity, stability, and site-specific yield traits. Only SNPs that were simultaneously detected by the three methods were retained. For each SNP-trait association, the corresponding -log10 (p)-values obtained from GEMMA, MLMM, and BLINK are presented. The SNP dDocent_Contig_31206_12 showed the strongest cross-method support: it exceeded the model-specific Bonferroni and FDR thresholds for STI and met the suggestive candidate threshold of P < 1 × 10^−4^ for MP, HM, and SYP_W in all three methods. All other associations shown in this table are cross-method-concordant suggestive signals rather than genome-wide-significant associations.

SNP	Trait	GEMMA	MLMM	BLINK
dDocent_Contig_31206_12	STI	6.24	6.34	8.52
	MP	5.28	5.44	5.56
	HM	4.73	4.87	4.88
	SYP_W	4.32	4.46	4.52
dDocent_Contig_39939_66	RPI	5.47	5.37	5.47
	TOL	4.47	4.35	4.42
dDocent_Contig_31857_122	RPI	4.39	4.31	4.39
	TOL	4.68	4.68	4.75
dDocent_Contig_40407_155	MP	4.51	4.06	4.36
	SYP_W	4.42	4.45	4.51
dDocent_Contig_50588_13	MP	4.83	4.57	4.68
	HM	4.65	4.23	4.21
dDocent_Contig_31206_69	MP	4.66	4.84	5.14
	HM	4.29	4.44	4.64
dDocent_Contig_74835_50	SSI	4.72	4.88	4.92
dDocent_Contig_39611_97	SYP_G	4.72	4.51	4.42
dDocent_Contig_1903_150	SYP_G	4.38	4.32	4.71
dDocent_Contig_11268_59	ASV	4.49	4.27	4.33

The genome-wide STI hit, dDocent_Contig_31206_12, was also the only SNP in the entire candidate set with a consistent, three-method-replicated association to more than three traits: beyond STI, it reached the candidate threshold in all three methods for SYP_W, MP, and HM, but not for SYP_G, RPI, YPI, LRR, TOL, ASV, or SSI (Fig 2).

**Fig 2:**
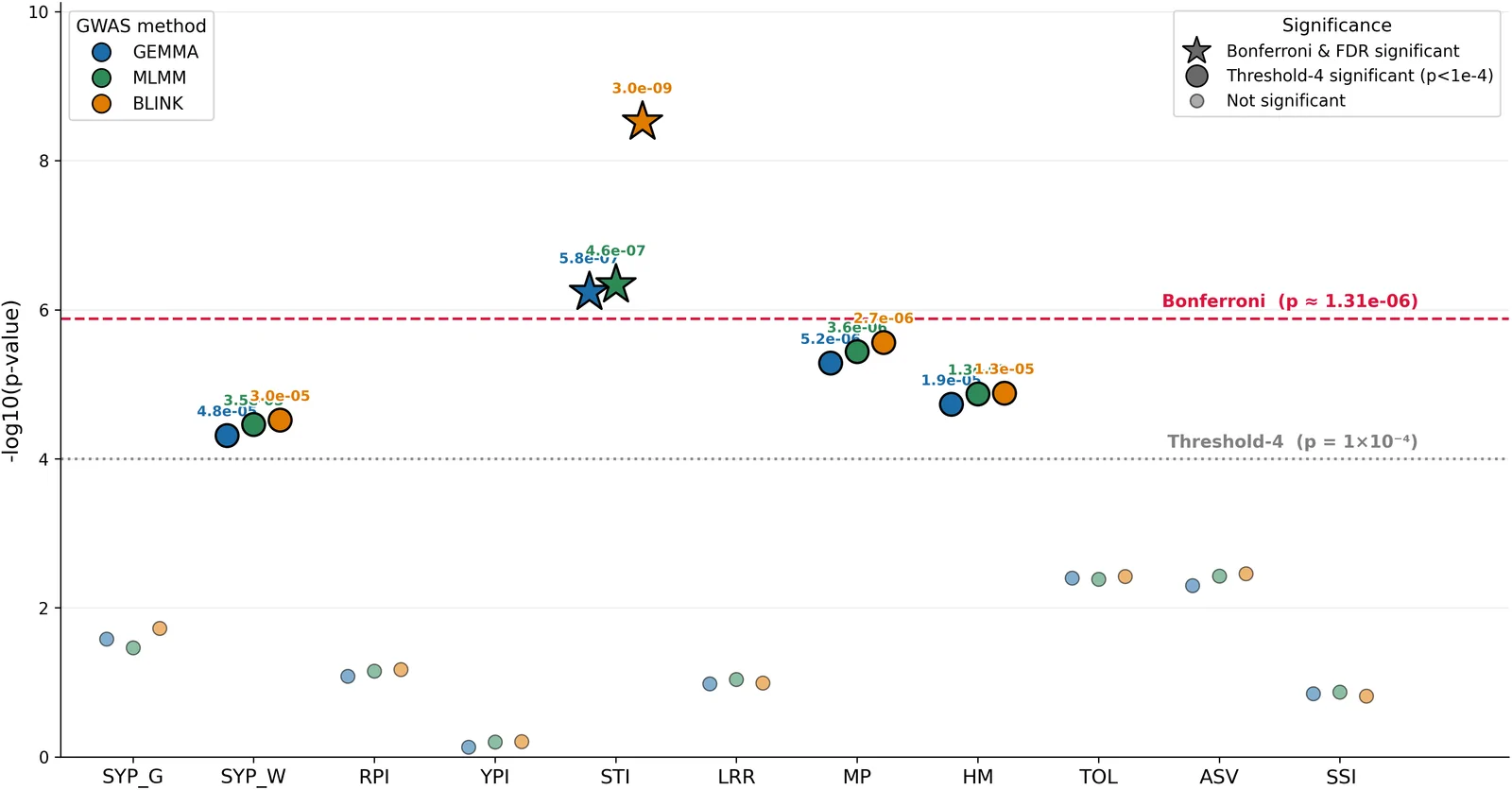
Association between the top pleiotropic SNP, dDocent_Contig_31206_12 and all 11 phenotypic indices, tested independently by three association mapping methods (GEMMA univariate LMM, MLMM, BLINK). Points are colored by method; stars denote SNPs surpassing both the Bonferroni and FDR (5%) thresholds, filled circles denote the relaxed threshold-4 cutoff (p < 1 × 10^−4^), and small pale circles denote non-significant associations. Exact p-values are printed above each significant point. The SNP reaches genome-wide significance in all three methods for STI (p = 5.8 × 10^−7^, 4.6 × 10^−7^, and 3.0 × 10^−9^ for GEMMA, MLMM, and BLINK, respectively) and clears the threshold-4 cutoff in all three methods for SYP_W, MP, and HM, but shows no association with the remaining seven traits.

At the relaxed candidate threshold (−log_10_(p) > 4, p < 1 × 10^−4^), and across trait–method combinations, zero to ten SNPs met the suggestive threshold (S2 Table), with generally consistent numbers across methods (e.g., RPI: 4 SNPs in all three methods; TOL: 5 SNPs in all three methods) and somewhat higher counts for BLINK in a few traits (e.g., MP: 6, 7, and 10 candidate SNPs for GEMMA, MLMM, and BLINK, respectively). Cross-referencing candidate SNPs across the three methods (Overlap_Summary) showed that the majority of candidates for RPI, YPI, TOL, HM, and SSI were replicated by all three methods, whereas SYP_G, SYP_W, STI, MP, and ASV showed partial concordance, with a subset of candidates detected by only two of the three methods (most often MLMM and BLINK jointly) and few or no method-exclusive candidates unique to GEMMA or MLMM alone.

Genome-wide screening for multi-trait consensus candidates (SNPs reaching the candidate threshold in all three methods for more than one trait) identified seven additional loci beyond dDocent_Contig_31206_12, each associated with exactly two traits: dDocent_Contig_30966_240 (SYP_W, TOL), dDocent_Contig_31206_69 (MP, HM), dDocent_Contig_31857_122 (RPI, TOL), dDocent_Contig_39939_66 (RPI, TOL), dDocent_Contig_40407_155 (SYP_W, MP), dDocent_Contig_45688_210 (RPI, TOL), and dDocent_Contig_50588_13 (MP, HM) (Fig 3). Notably, three of these seven loci co-associate with the same trait pair (RPI and TOL), and two co-associate with MP and HM, consistent with the mathematical relationships linking these derived indices to the same underlying yield measurements (SYP_G, SYP_W). dDocent_Contig_31206_12 remains distinct from this group both in the number of traits it associates with (four, versus two for all other multi-trait loci) and in being the only locus among them to also cross the strict genome-wide significance threshold, making it the strongest and most broadly pleiotropic candidate locus identified in this panel.

**Fig 3:**
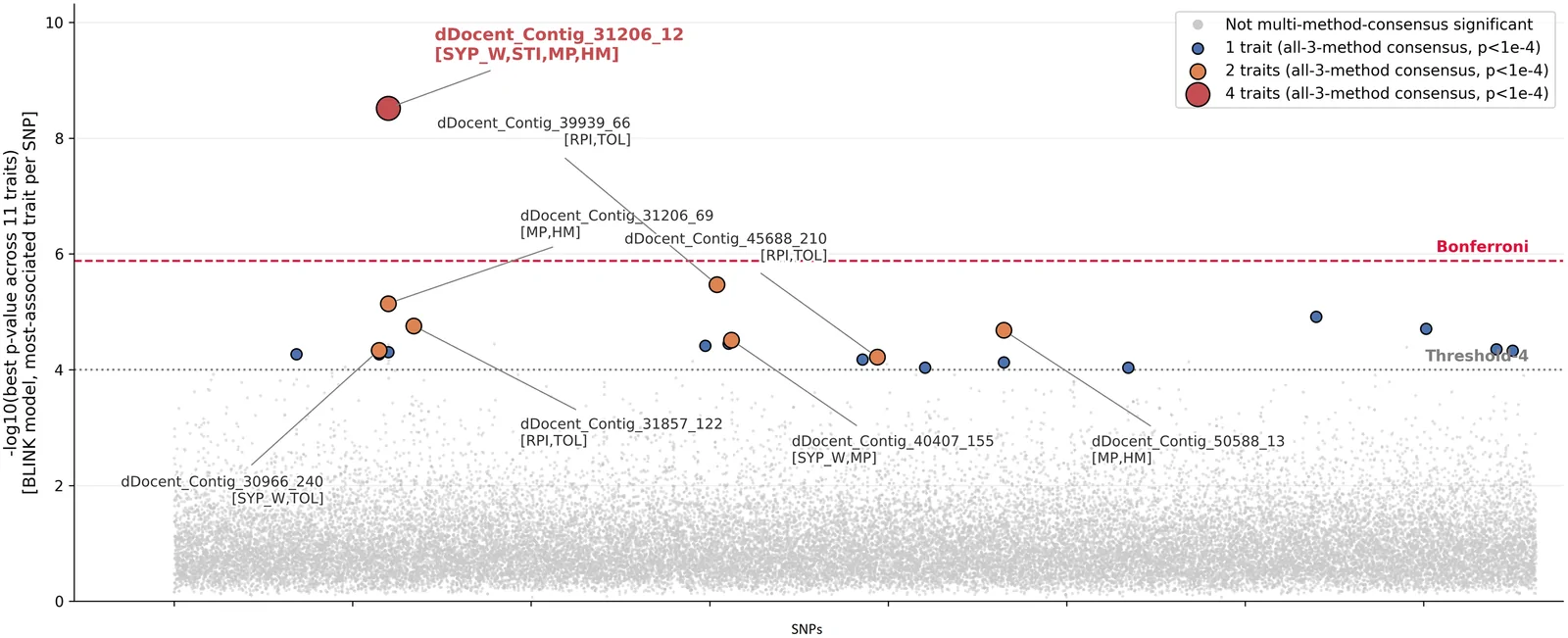
Genome-wide distribution of the 38,142 SNPs (no physical reference genome was available for this panel) against each SNP’s strongest association (−log10 p) across the 11 traits, using the BLINK model. Grey points are SNPs that do not reach the threshold-4 cutoff (p < 1 × 10^−4^) concurrently in all three association mapping methods (GEMMA, MLMM, BLINK) for any trait; colored points denote SNPs that do, sized and colored by the number of distinct traits for which this three-method consensus holds (blue = 1 trait, orange = 2 traits, red = 4 traits). Each colored SNP is labelled with its identifier and associated trait(s). One locus, dDocent_Contig_31206_12, stands out as the only SNP associated with more than two traits (SYP_W, STI, MP, and HM) and is the sole SNP in the panel to also cross the Bonferroni threshold (dashed red line); seven additional loci show two-trait consensus associations, most pairing traits that are mathematically or biologically related (e.g., RPI with TOL, MP with HM).

### Multivariate association analysis

The GEMMA multivariate mixed linear model (mvLMM; SYP_G + SYP_W, 2 d.f. Wald test) identified dDocent_Contig_31206_12 as strongest multivariate association signal (Table 3; Fig 4; S3 Table), with a multivariate association of p = 8.29 × 10^−6^ (−log_10_p = 5.08). The allele was associated with positive effects on seed yield in both environments (β_Giza = +7.96; β_Wadi = +33.69). A closely linked marker within the same genomic region, dDocent_Contig_31206_69, showed a similar pattern of positive effects (β_Giza = +7.40; β_Wadi = +32.43) and ranked as the third strongest multivariate signal

**Table 3:** Significant SNPs identified by multivariate genome-wide association analysis (mvGWAS) in GEMMA for the jointly analyzed traits SYP_G and SYP_W. The table reports SNP identifiers, estimated marker effects (β) for each trait, and the overall multivariate significance expressed as −log10(p). The β coefficients represent the expected change in trait value associated with each additional copy of allele 1 relative to the reference allele; positive values indicate an increase in the trait, whereas negative values indicate a decrease. Because the fitted genetic and residual covariance decomposition was unstable, the phenotype-specific beta estimates are presented descriptively and should not be interpreted as precise independent site-specific effects.

SNP	Β-SYP_G	β-SYP_W	-log10(p)
dDocent_Contig_31206_12	7.96	33.69	5.08
dDocent_Contig_50588_13	−9.42	−28.28	4.81
dDocent_Contig_31206_69	7.40	32.43	4.74
dDocent_Contig_1903_150	14.30	10.70	4.53
dDocent_Contig_39611_97	−27.82	11.49	4.51
dDocent_Contig_31857_122	−6.24	29.95	4.40
dDocent_Contig_117_103	18.58	−4.25	4.21
dDocent_Contig_61204_281	−9.96	−24.15	4.21
dDocent_Contig_10066_205	15.13	2.57	4.17
dDocent_Contig_40407_155	−3.67	−34.13	4.10
dDocent_Contig_3191_220	4.03	37.14	4.06

No SNP exceeded the stringent Bonferroni-adjusted genome-wide significance threshold (0.05/37,885 = 1.32 × 10^−6^; − log_10_p = 5.88). Nevertheless, genomic control was well calibrated (λGC = 0.995).

**Fig 4:**
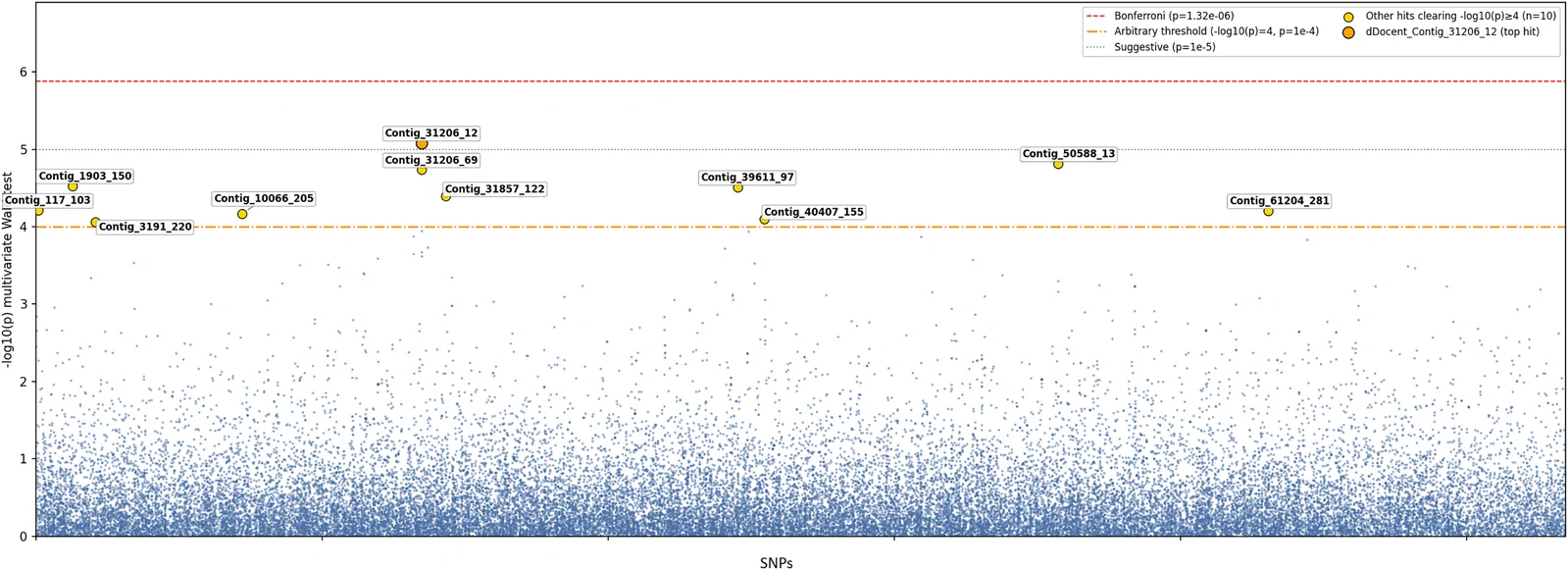
Manhattan plot, full-genome multivariate GEMMA LMM (SYP_G + SYP_W, 37,885 markers). All points share a single colour (contig identity is not colour-coded). Red dashed line: Bonferroni threshold (p = 1.32 × 10^−6^). Orange dash-dot line: arbitrary candidate threshold (-log_10_(p) = 4, p = 1 × 10^−4^), following the convention used in Abd El-Wahab et al. (2023) and Mosalam et al. (2025) for this research line. Gold/orange points and labels: the 11 markers clearing this arbitrary threshold (S2 Table), each annotated with its SNP ID; dDocent_Contig_31206_12, the top hit, is distinguished in darker orange.

Using the more permissive candidate threshold of −log_10_(p) ≥ 4 (p ≤ 1 × 10^−4^), 11 SNPs (0.03% of the 37,885 markers tested) were identified as suggestive multivariate associations. These included the robust locus dDocent_Contig_31206_12, its linked variant dDocent_Contig_31206_69, and dDocent_Contig_39611_97, all of which were also supported by univariate association analyses. The descriptive beta estimates had the same sign for some loci and opposite signs for others; these patterns require cautious interpretation because the multivariate covariance decomposition was unstable. For example, dDocent_Contig_50588_13 and dDocent_Contig_61204_281, whose alleles were associated with reduced seed yield under both environments (negative β values for SYP_G and SYP_W). In contrast, markers such as dDocent_Contig_39611_97, dDocent_Contig_31857_122, and dDocent_Contig_117_103 displayed opposite effect directions between environments.

### Contig 31206 locus structure

The five markers on ddRAD contig 31206 were in high pairwise linkage disequilibrium (r² = 0.68–0.95), and 91.6% of accessions carried one of just two multilocus haplotypes. Conditional analysis on the lead marker (Fig 5) reduced all four flanking markers to non-significance for STI (conditional p = 0.58–0.85), supporting a single represented locus rather than multiple independent signals. Leave-one-accession-out re-analysis across all 107 accessions kept the association within a narrow, consistently significant range (p = 1.4 × 10^−7^ to 3.4 × 10^−6^), and simultaneous exclusion of three named high-yield accessions (G15, G98, G109) weakened but did not eliminate the association (p = 9.6 × 10^−5^).

**Fig 5:**
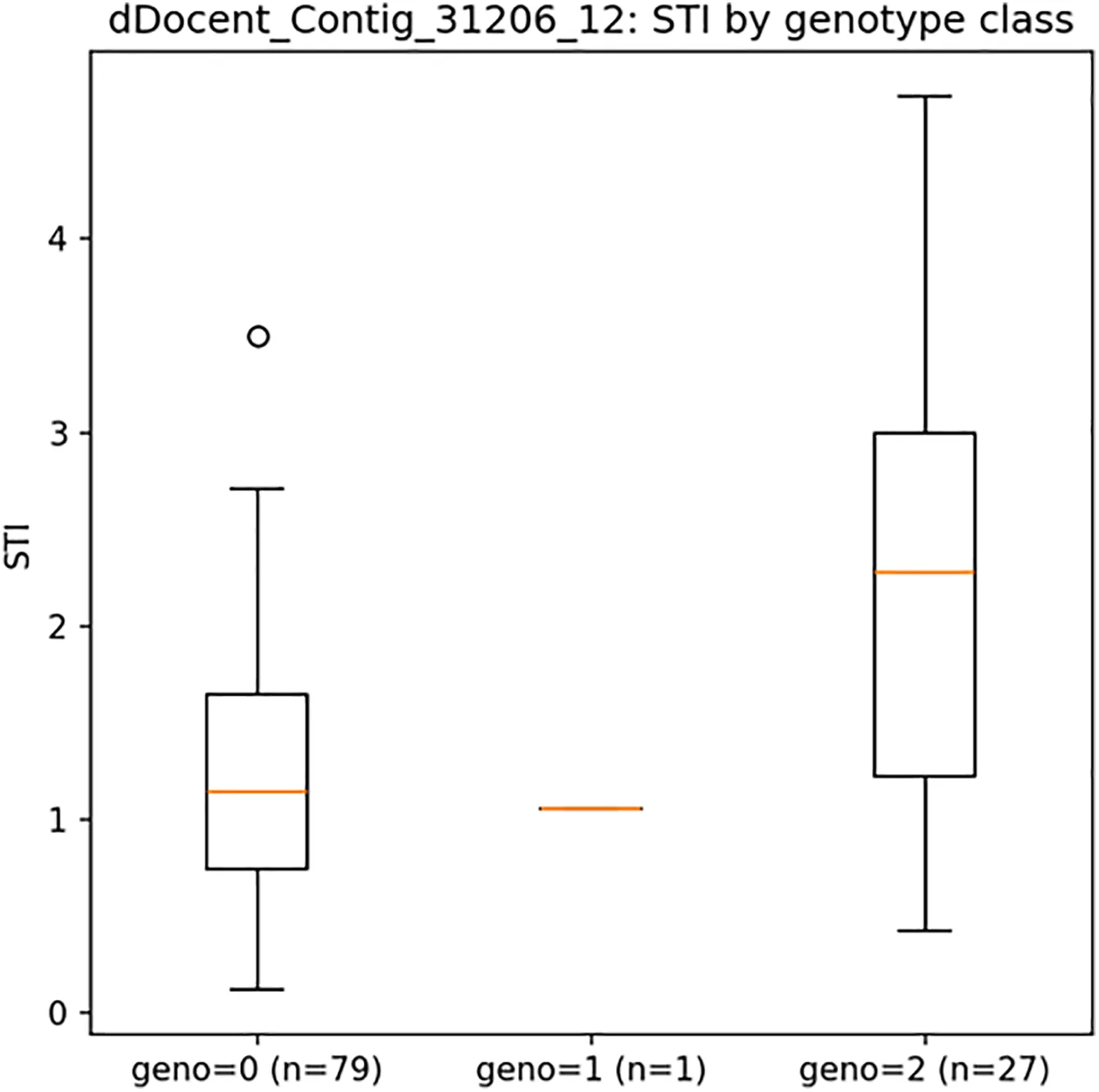
Effect of the lead locus, dDocent_Contig_31206_12, on stress tolerance index (STI). Boxplots show the distribution of STI values for the three observed genotype classes at the lead SNP. Accessions homozygous for the alternative allele had higher STI values than accessions homozygous for the reference allele. Because the heterozygous class contained only one accession, the mode of allele action could not be evaluated reliably.

### Comparative homology analysis of candidate loci

Systematic local BLAST+ searches of all 32 candidate SNPs against three reference legume genomes (*Medicago truncatula, Trifolium repens, Trifolium pratense*) resolved the candidate set into three categories (Table 4). Two SNPs, both associated with STI, showed copy-number and locus-clustering patterns consistent with genuine single- or low-copy nuclear genes across all three genomes: dDocent_Contig_33805_254 (cation/H⁺ antiporter 15), for which copy number tracked ploidy exactly (1–2–1 loci in diploid–tetraploid–diploid), and dDocent_Contig_46121_295 (importin subunit beta-1), recovered as a small, syntenic tandem gene family (2, 4, and 3 clustered loci respectively) in all three genomes.

**Table 4:** Candidate genes supported by three-genome comparative homology validation (*Medicago truncatula, Trifolium repens, Trifolium pratense*). Both supported candidates are associated with the Stress Tolerance Index (STI).

SNP	Trait (method, p)	Putative gene	M. truncatula	T. repens (allotetraploid)	T. pratense
dDocent_Contig_33805_254	STI (BLINK, p = 5.8 × 10^−5^)	Cation/H⁺ antiporter 15	1 locus, chr. 3; 94.1% identity, full 101 bp, E = 5.8 × 10^−38^	2 loci — chr03_Occ and chr03_Pall (one per subgenome); 83–88% identity, full length	1 locus, LG7; 84.2% identity, full 101 bp, E = 9.6 × 10^−24^
dDocent_Contig_46121_295	STI (MLMM, p ≈ 8.6 × 10^−5^)	Importin subunit beta-1	2 tandem loci, ~ 17.6 kb apart, chr. 3	4 loci = 2 tandem pairs × 2 subgenomes (chr03_Occ, chr03_Pall)	3 loci clustered within ~480 kb, LG2

By contrast, two of the strongest association signals in the dataset were identified as confirmed repetitive elements rather than single-copy genes. dDocent_Contig_31206 (the top STI/SYP_W signal; SNPs 12, 69, 87, 124, 144) produced BLASTn hits to multiple independent *M. truncatula* loci on two chromosomes, dispersed moderate-identity hits across seven or more chromosome arms in *T. repens*, a taxonomically incongruent hit distribution (a gymnosperm, Cannabis sativa, and an unclassified environmental clone), and a translated-protein CCHC-type zinc-knuckle plus coiled-coil architecture characteristic of retrotransposon Gag-like nucleocapsid proteins — a confirmed high-copy or dispersed repeat family rather than a coding gene. dDocent_Contig_39939_66 showed an even more extreme pattern: 327 distinct strong genomic loci in *M. truncatula*, 52 in *T. repens*, and 7 in *T. pratense*, confirming a high-copy repeat family in all three genomes. One further SNP (dDocent_Contig_10066_205, SYP_G) initially appeared single-copy in *M. truncatula* but showed the same dispersed, moderate-identity pattern as the confirmed repeats when tested in *T. repens*, and is accordingly not reported as a validated candidate. None of the candidate sequences produced a strong match to the available fenugreek plastid or mitochondrial genomes, supporting, but not definitively proving, a nuclear origin.

## Discussion

The study was designed to characterize spatial environmental contrast within a single season. Consequently, the observed genotype responses and marker associations cannot distinguish genotype × location from genotype × year effects, and their temporal stability requires validation across additional seasons. Within this framework, the two environments evaluated in this study exhibited contrasting effects on yield performance and provided insight into the nature of environmental adaptation in fenugreek.

### Wadi El-Natrun as an environmental responsiveness-revealing environment and evidence for G × E

The performance patterns observed in this study, including higher mean yield in Wadi El-Natrun, a high proportion of accessions with RPI > 1, and substantial phenotypic variation, are consistent with previously reported phenotypic analyses of this germplasm panel [8] but are here reinterpreted in a genomic and adaptation-focused context, demonstrating that Wadi El-Natrun functions as an adaptation-revealing environment rather than a uniformly stress-imposing one.

The weak correlation between SYPG and SYPW is consistent with substantial genotype reranking and crossover G × E patterns across environments, indicating that genotype rankings are environment-specific and that adaptive variation remains partially hidden under favourable conditions [3,25]. This pattern highlights the importance of evaluating germplasm across contrasting production environments to reveal adaptive capacity and provides a biological basis for identifying SNPs associated with environmental responsiveness.

These findings support the concept that environmental contrast, rather than stress intensity alone, plays a central role in revealing adaptive genetic variation and provide a robust biological framework for the genomic dissection of adaptation performed in this study.

### Phenotypic robustness and genetic architecture of adaptation

Absolute productivity varied markedly between the two environments, and genotype rankings showed substantial environment dependence. Similar patterns have been reported in rapeseed, wheat, and rice, where genotype performance changes considerably across contrasting conditions despite appreciable genetic control [26–29]. These observations emphasize the importance of evaluating germplasms directly in target production environments rather than inferring adaptation from performance in a single environment.

The present findings support the interpretation of environmental responsiveness as an emergent property of G x E rather than a fixed genotype characteristic [30,31]. Within the current panel, marker coverage, and significance thresholds, most adaptation-index associations did not overlap the yield-associated loci reported previously in this panel [8]. One exception was dDocent_Contig_39939_66, which was previously associated with seed yield and showed suggestive associations with RPI and TOL across all three mapping methods in the present study. Together with the near-zero correlation between SYP_G and SYP_W (r = 0.107), this limited overlap suggests that environmental responsiveness represents a partially differentiated but environmentally structured component of yield performance rather than an independent biological process [3,32]. In addition, reliance on the FDR and nominal suggestive thresholds necessarily increases the probability that some reported associations are false positives; with N = 107, this risk is higher than in larger-panel, and suggestive-tier loci in particular should be regarded as hypothesis-generating rather than confirmed until independently validated.

Nevertheless, the limited detected overlap should not be interpreted as evidence that productivity and environmental responsiveness have independent genetic architectures. The modest population size and reduced-representation marker coverage may have limited the detection of shared small-effect loci. Moreover, RPI and YPI are mathematical functions of SYP_G and SYP_W. Consequently, a locus with differential effects on yield in the two environments may generate an association with an adaptation index without necessarily representing an independent adaptation pathway. The association results are therefore more appropriately interpreted as evidence of environment-specific genomic effects on yield than as proof of genetically autonomous adaptation mechanisms.

Treating RPI and YPI as separate analytical phenotypes nevertheless provides useful information about proportional response and the magnitude of yield change between environments. Comparable yield- and stress-index approaches have been used to identify genomic regions associated with drought responsiveness in wheat [12]. In the present study, however, the close phenotypic relationships among SYP_W, RPI, and YPI indicate that these indices primarily capture environment-dependent yield expression. This interpretation provides a more cautious framework for distinguishing responsiveness from joint productivity across environments.

Multivariate association mapping offered a complementary means of detecting loci with coordinated effects on related phenotypes. The joint SYP_G + SYP_W analysis identified several candidate SNPs, some of which did not reach the suggestive threshold in either corresponding univariate analysis. This result illustrates how multivariate models can improve sensitivity to coordinated, moderate effects that may remain undetected when related traits are analysed separately [33,34]. The stronger suggestive signals observed for RPI than for YPI may partly reflect their different statistical distributions. YPI is bounded between zero and one, which can compress phenotypic variation near its limits and potentially reduce association-mapping power relative to an unbounded response measure such as RPI [35,36].

From a breeding perspective, the lack of detected overlap between yield- and responsiveness-associated loci suggests that selection based solely on yield potential in one environment may not capture the alleles contributing to differential performance across environments. Productivity and environmental responsiveness may therefore need to be considered jointly, while recognizing that the present evidence does not establish them as completely independent breeding targets. Validation in additional environments, seasons, and independent germplasm will be required to determine whether the observed locus-specific effects are temporally stable and transferable across populations. Larger mapping populations would also be valuable for further evaluating whether additional loci with shared effects on yield and environmental responsiveness exist but were undetected here because of limited statistical power

Comparative genomic analysis further showed that candidate-locus interpretation must distinguish the statistical strength of an association from confidence in its functional annotation. Searches against three legume reference genomes identified two loci with sequence and copy-number patterns consistent with genuine single- or low-copy nuclear genes: a cation/H⁺ antiporter homolog associated with dDocent_Contig_33805_254 and an importin subunit beta-1 homolog associated with dDocent_Contig_46121_295. Both loci were associated with STI rather than directly with RPI or YPI. Their functions nevertheless provide biologically plausible hypotheses. Cation/H⁺ antiporters contribute to cellular ion and pH homeostasis under osmotic and ionic stress, whereas importin-beta proteins mediate the nucleocytoplasmic transport of transcription factors and other regulatory proteins. These annotations strengthen the biological plausibility of the loci, but they do not establish causality and require validation in independent populations and functional studies [5].

The homology analysis also identified an important limitation of reference-free, ddRAD-based association mapping. Two of the strongest statistical signals corresponded to repetitive elements rather than single-copy genes. dDocent_Contig_31206_12, the only SNP that exceeded the strict genome-wide significance threshold and the most broadly associated locus in the panel, displayed sequence characteristics consistent with a retrotransposon Gag-like nucleocapsid element. Similarly, dDocent_Contig_39939_66 matched numerous dispersed positions across all three reference genomes, supporting its classification as a high-copy repeat. These classifications do not invalidate the marker-trait associations. In particular, the linkage-disequilibrium, conditional, and leave-one-accession-out analyses support the statistical robustness of the Contig 31206 association. Nevertheless, the findings prevent these sequences from being interpreted as specific causal genes. They may represent anonymous markers in linkage disequilibrium with functional variants, but their repetitive genomic context could complicate assay development and transferability.

Collectively, the candidate-locus results do not yet support a single mechanistic model of environmental responsiveness in fenugreek. The two comparatively supported loci implicate ion and pH homeostasis and nucleocytoplasmic transport, whereas the motif-based regulatory candidates remain unconfirmed and two statistically prominent signals correspond to repetitive elements. Targeted sequencing, gene-expression analysis, validation in independent germplasm, and multi-season field evaluation will therefore be required before specific genes or pathways can be assigned causal roles in environment-dependent yield performance.

### Alternative performance strategies across environments

The distinct genetic architecture identified for adaptation traits raises the question of how adaptation is expressed phenotypically within the germplasm collection. Examination of accession-level performance revealed contrasting adaptive strategies represented by highly responsive and highly stable genotypes.

The mutual exclusivity of Adapted High Yielders and Stable High Yielders (overlap N = 0) highlights contrasting performance strategies within the germplasm panel. Part of this pattern arises from the mathematical relationship between the adaptation indices themselves. When SYP_W > SYP_G, YPI can be expressed as YPI = 1 − (1/RPI), making a positive correlation between RPI and YPI inherently expected; accordingly, the observed correlation (r = 0.710) should not be interpreted solely as evidence of a biological trade-off.

Nevertheless, the separation of highly responsive and highly stable genotypes remains biologically and practically meaningful. Accessions such as G41 (Beheira; SYP_G = 172.4 g, SYP_W = 172.6 g, YPI = 0.001) represent a constitutive buffering strategy characterised by nearly identical performance across environments. In contrast, G15 (RPI = 3.629, SYP_W = 409.0 g) and G27 (RPI = 4.560) exemplify highly responsive genotypes that achieve superior performance specifically under Wadi El-Natrun conditions. These contrasting phenotypic profiles represent alternative breeding targets rather than mutually exclusive biological categories [37,38]. The coexistence of these performance profiles suggests that the panel contains multiple phenotypic response strategies, although their physiological and genetic bases remain unresolved [3,30].

The proposed cross G41 × G15, therefore, represents an opportunity to explore whether favourable alleles underlying environmental stability and environmental responsiveness can be combined within segregating populations. G109 (Beni Suef; SYP_G = 149.9 g, SYP_W = 330.4 g, RPI = 2.204) is particularly notable because it combines strong performance in both environments while maintaining relatively balanced adaptation characteristics, making it one of the most promising candidates for immediate breeding use.

### Breeding recommendations

The integrated phenotypic and genomic results support the following tiered strategy:

- Direct Wadi El-Natrun cultivation: G15 and G98 (Qena; SYP_W ≥ 407 g, RPI ≥ 3.60), G24 (Beni Suef; RPI = 4.53), G53 (Ismailia; SYP_W = 312.7 g), and G31 (Faiyum; RPI = 2.60) emerge as priority candidates for multi-site validation.
- Dual-environment cultivation: G109 (Beni Suef; SYP_G = 149.9 g, SYP_W = 330.4 g, RPI = 2.20) and G107 (Minya; SYP_G = 130.7 g, SYP_W = 279.9 g) combine competitive performance in both environments. G41 (Beheira; YPI = 0.001, SYP_G = 172.4 g) is the highest-priority accession where cross-environment yield consistency is paramount.
- Crossing programme: Priority crosses G41 × G15 (constitutive stability × high Wadi responsiveness) and G109 × G27 (dual elite × high RPI) are suggested as promising combinations. F_2_ selection in the G41 × G15 cross could target progeny with SYP_G ≥ 120 g, RPI ≥ 2.0, and YPI ≤ 0.50 to combine yield stability with Wadi performance. Multi-location validation across at least two seasons would strengthen the assessment of these crosses.

## Conclusion

Results suggest substantial genotype reranking consistent with G × E contributing to adaptation.

Wadi El-Natrun functioned as a contrasting, adaptation-revealing environment and produced genotype reranking consistent with crossover G × E. The near-zero correlation between environments demonstrated that performance under Giza conditions alone provides little information about performance under contrasting production environments, emphasizing the importance of direct evaluation under target conditions. Association mapping showed limited overlap between previously reported yield loci and the current adaptation-index associations, suggesting partially differentiated but interconnected genomic effects on yield and environmental responsiveness.

Taken together, the phenotypic and genomic evidence suggest that adaptation in fenugreek is best viewed as an emergent property of G × E rather than a fixed genotype characteristic. Consequently, breeding programmes targeting diverse production systems should combine direct evaluation in target environments with genomic approaches that explicitly address adaptive responsiveness in addition to yield potential. These findings contribute to the growing body of evidence that adaptation and yield represent partially distinct but interconnected breeding targets shaped by environment-specific yield responses.

## Supporting information

S1 Table Environmental characterization of the two experimental sites. Soil physical and chemical properties and irrigation water quality data of the Giza and Wadi El-Natrun field sites. EC: electrical conductivity; ET_0_: reference evapotranspiration. Values for soil texture and nutrients are means of composite samples. Climatic parameters represent seasonal averages during the cropping period (January–June).
(XLSX)

S2 Table Results from univariate association scans for SYP_G, SYP_W, RPI, YPI, STI, LRR, MP, HM, TOL, SSI, and ASV using GEMMA, MLMM, and BLINK. The workbook reports SNPs meeting at least one of the following criteria within a trait-model scan: the model-specific Bonferroni threshold, Benjamini-Hochberg FDR q ≤ 0.05, or the exploratory candidate threshold of P < 1 × 10^−4^. Summary_Counts reports the number of detected SNPs, while Overlap_Summary and Overlap_SNP_Detail report concordance among methods.
(XLSX)

S3 Table Results from the multivariate GEMMA analysis of SYP_G and SYP_W. The table reports the 11 SNPs with joint two-degree-of-freedom Wald-test P ≤ 1 × 10^−4^, including SNP identifier, contig, within-contig position, allele coding, allele frequency, phenotype-specific effect estimates, variance-covariance estimates, joint p-value, and −log_10_(P). No SNP exceeded the multivariate Bonferroni threshold of P ≤ 1.32 × 10^−6^.
(XLSX)
